# Prepubertal nutrition alters Leydig cell functional capacity and timing of puberty

**DOI:** 10.1371/journal.pone.0225465

**Published:** 2019-11-21

**Authors:** Ravinder Anand-Ivell, Colin J. Byrne, Jonas Arnecke, Sean Fair, Pat Lonergan, David A. Kenny, Richard Ivell

**Affiliations:** 1 School of Biosciences, University of Nottingham, Sutton Bonington, United Kingdom; 2 Animal and Bioscience Department, Teagasc, Dunsany, Ireland; 3 School of Agriculture and Food Science, University College Dublin, Dublin, Ireland; 4 Laboratory of Animal Reproduction, Department of Biological Sciences, University of Limerick, Limerick, Ireland; University of Tasmania, AUSTRALIA

## Abstract

Leydig cell functional capacity reflects the numbers and differentiation status of the steroidogenic Leydig cells in the testes and becomes more or less fixed in early adulthood with the final establishment of the hypothalamo-pituitary-gonadal (HPG) axis after puberty. Factors influencing Leydig cell functional capacity and its role in puberty are poorly understood. Using a bovine model of dairy bulls fed four different nutritional regimes from 1 month to 12 months, and applying circulating Insulin-like peptide 3 (INSL3) as an accurate biomarker of Leydig cell functional capacity, showed that a high plane of nutrition in the first 6 months of life, but not later, significantly increased INSL3 in young adulthood. Moreover, INSL3 concentration at 4 months indicated a marked differential in early feeding regime and correlated well (negatively) with the timing of puberty, as reflected by the age in days for the first production of an ejaculate with >50 million sperm and >10% forward motility, as well as with testis size at 18 months. Reversing the diet at 6 months was unable to rectify the trend in either parameter, unlike for other parameters such as testosterone, body weight, and scrotal circumference. This study has shown that early prepubertal nutrition is a key factor in the development of Leydig cell functional capacity in early adulthood and appears to be a key driver in the dynamic progression of puberty.

## Introduction

The Leydig cells of the testes are responsible for producing the major sex steroids throughout life and are essential for supporting sperm production [1]. Their numbers and differentiation status (functional capacity) are established during puberty and change little across the lifespan, thereby correlating also with testosterone production and morbidity in older age [2]. Besides testosterone and androstenedione, the Leydig cells also make and secrete large amounts of the constitutive peptide hormone INSL3 (insulin-like peptide 3) which has become an important new biomarker uniquely reflecting Leydig cell functional capacity [2], and which may vary up to ten-fold in adult men [3]. However, relatively little is known about the early life factors which influence the relative rates of Leydig cell proliferation and differentiation, and hence Leydig cell functional capacity in the young adult.

In order to explore the effect of nutrition on this parameter, we have made use of a bovine model in which food intake in the prepubertal period has been carefully controlled [4]. The model was developed because application of modern genetic selection algorithms to young calves, rather than waiting for actual sire performance, is putting pressure on the cattle industry to accelerate the growth and sexual maturation of bulls to deliver qualitatively and quantitatively adequate semen as early as possible. For modern *Bos taurus* breeds, puberty as defined by the attainment of 50 million sperm in an ejaculate with >10% forward motility [5] occurs at 8–10 months of age, and in tropical breeds, including *B*. *indicus*, may be as late as 15 months [6]. Such early semen may not prove adequate in terms of fertilizing potential nor overall quantity [7,8]. There is therefore a need for a better understanding of the physiological processes involved as well as criteria by which to monitor optimal physiological and sexual development, thus allowing collection of semen with consistent and reliable quality and quantity as early as possible. Such criteria could be used to predict the timing of puberty and understand the processes being modulated by, for example, altering management and feeding regimes.

Sexual development in the bull can be broadly divided into two phases: the prepubertal phase occupies approximately the first 6 months of life; and the peri-pubertal phase from 6 to 12 months [9,10]. As in all mammals, these phases are governed primarily by the hormones of the HPG (hypothalamo-pituitary-gonadal) axis [11]. In the bovine, modest levels of pituitary Follicle Stimulating Hormone (FSH) promote the proliferation and then maturation of testicular Sertoli cells from about 5 weeks through to about 32 weeks, whereas the steroidogenic Leydig cells undergo a rapid proliferation and maturation phase between 5 and 28 weeks, largely under the influence of a prepubertal surge of Luteinising Hormone (LH) evident between 10 and 20 weeks [9]. In contrast, spermatogenesis commences only around week 20 [9]. The appearance of sperm in the ejaculate as well as scrotal circumference both reflect the process of spermatogenesis: in the mature testis, Leydig cells comprise usually only about 10% of the cell volume [12] and Sertoli cells even less [4], though this may vary between species, with the remainder mostly dedicated to sperm production. Sertoli cells support and nourish the differentiating germ cells, whose number ultimately reflects the quality and quantity of the Sertoli cells. Leydig cells make both local paracrine factors essential for the healthy development of the seminiferous epithelium, including Sertoli cells, as well as androgens, such as testosterone, required for spermatogenesis and for general growth and metabolism of the animal including its appropriate behavioural development.

To date, circulating testosterone and/or its relationship to the gonadotropin LH has been used as a measure of Leydig cell development [1]. Testosterone and LH are challenging to measure accurately due to high technical and biological variance. Both are secreted in response to GnRH in a pulsatile manner, which is particularly dramatic during puberty. In contrast, INSL3 is a major secretory product of the steroidogenic Leydig cells of the testes in all mammals so far investigated [2,13], but unlike testosterone does not appear to show marked diurnal or intra-individual variation [13,14]. It is produced and secreted constitutively into the bloodstream, in a manner which appears to be acutely independent of the hormones of the HPG axis [15,16]. INSL3 production only appears to be dependent upon the number and stage of development of the individual Leydig cells within the testes, and is not made by any other organ in the male [2,13,17]. However, its expression may be influenced by LH in the long-term since this influences the differentiation of the Leydig cells, and high pulses of exogenous LH may also affect its production [18]. Findings from humans and rodents [2,19] support the view that INSL3 is an excellent biomarker for Leydig cell functional capacity (i.e. the residual potential to make androgenic steroids and other factors) and could represent an important parameter with which to assess pubertal progression in bull calves, especially during the important prepubertal phase.

Whilst most information concerning INSL3 expression in the male pertains to rodents or humans, limited findings from bulls fully support the above interpretation. Northern blot of multiple male tissues shows that the testis is the only male tissue to express INSL3 mRNA [20] and, when castrated, steers indicate no circulating INSL3 above the non-detectable level ([21] and Anand-Ivell R, unpublished). Within the bovine testis both in situ mRNA hybridization [20] as well as immunohistochemistry using different antibodies [22,23] confirm that only Leydig cells express INSL3. Moreover, circulating INSL3, as in the present article, correlates closely to the growth of the testes between birth and puberty (present article; [24]).

The present study evaluated the expression of INSL3 during pubertal progression in male Holstein-Friesian calves which had been closely monitored for a series of physical and hormonal parameters under four different feeding regimens [4,25]. For the first 6 months of life, calves were fed either a relatively low plane of nutrition (Lo) or a high plane of nutrition (Hi); these two groups were further subdivided for the peri-pubertal period between 6 and 12 months, the calves in each group being provided either a continuation of the high or low feeding regimen, or their reversal, creating four groups of animals referred to as Hi-Hi, Hi-Lo, Lo-Hi, and Lo-Lo, respectively [4,25]. The study used a well-validated homologous time-resolved fluorescent immunoassay (TRFIA) to measure INSL3 in peripheral serum [22,26], which was further optimized for the current application. The results indicate that INSL3 expression is markedly influenced by nutritional status and offers an additional and very useful parameter of low variance with which to map pubertal trajectory. In particular, the importance of the prepubertal period for the establishment of a persistent high Leydig cell functional capacity was clearly evident.

## Materials and methods

### Animals and procedures

Details of the study protocol have been described previously [4]. All procedures on animals were carried out under license from the Irish Department of Health and Children (license no. B100/4516), and followed the Cruelty to Animals Act (Ireland 1876, as amended by EC regulations 2002 and 2005) and the EC Directive 86/609/EC. All calves were sourced from commercial dairy farms and housed at Teagasc Grange, Animal and Grassland Research Centre, Dunsany, Co. Meath, Ireland. Calves were group housed on sawdust bedding in well-ventilated, purpose-built calf housing. Briefly, autumn-born Holstein-Friesian male calves (n = 83) were blocked according to age, body weight, sire and farm of origin and assigned either a high (Hi) or low (Lo) plane of nutrition until 6 months of age. Those assigned to Hi (n = 37) or Lo (n = 46) were provided 1200 g or 450 g milk replacer, respectively. The former were fed concentrate *ad libitum*, the latter only to a maximum of 1 kg per day. Calves were fed individually using an electronic feeding system (Vario, Forster-Technik, Engen, Germany) until weaning. Hay as roughage and water were offered *ad libitum* to all calves. They were penned according to treatment and turned out to pasture at 6 months, with rotational grazing in their respective treatment groups. Animals either remained on the same diet or were switched to the opposite diet until onset of puberty, creating four treatment groups: Hi-Hi (n = 19), Hi-Lo (n = 18), Lo-Hi (n = 22), and Lo-Lo (n = 24). In order to determine the onset of puberty, electroejaculation commenced every two weeks at a scrotal circumference of 24 cm. Attainment of puberty was defined here as the first of two successive electro-ejaculates with >50 million sperm and >10% progressive motility [5]. After puberty, all bulls were fed a moderate plane of nutrition [4] until slaughter at 18 months, when both testes were removed and weighed together (paired testis weight). Animals were not subjected to any procedures that required administration of anaesthetic or analgesic and at 18 months were euthanized using a captive bolt, followed by exsanguination at Eurofarm Foods, Duleek, Co. Meath (EU factory approval number: EC297).

### Assayed parameters

Monthly blood samples were collected from the jugular vein into either Li-plasma tubes (for Insulin-like Growth Factor 1, IGF1) or allowed to clot overnight for serum (gonadotropins, total testosterone, INSL3). IGF1 was measured by validated radioimmunoassay (RIA) following acid-ethanol extraction [27]; intra- and inter-assay coefficient of variation (CV) were 12.5, 6.6, and 5.1% and 13.7, 8.4, and 9.6% for low, medium and high values, respectively, with a limit of detection (LOD) of 4 ng/ml. Serum FSH was measured by the method of Crowe et al. [28] with an LOD of 0.05 ng/ml; intra- and inter-assay CV were 8.7, 7.2, and 9.3% and 15.8, 19.7, and 9.5% for low, medium, and high values, respectively. Serum LH was determined by the method of Cooke et al. [29] with a polyethylene glycol separation step [25]. The LOD was 0.05 ng/ml, and intra- and inter-assay CV were 9.1, 18.2, and 7.2% and 2.5, 4.6, and 2.4% for low, medium, and high values, respectively. Total serum testosterone was determined by solid-phase RIA (DIAsource Immunoassays, Louvain-la-Neuve, Belgium) as previously described [30]. The LOD was 0.1 ng/ml, and intra- and inter-assay CV were 12.1, 10.0, and 9.0% and 12.5, 8.2, and 9.5% for low, medium, and high values, respectively.

INSL3 was measured in serum using a validated, species-specific time-resolved fluorescent immunoassay (TRFIA), as described previously [22,26,31]. This was modified slightly by three-fold sample dilution to accommodate the higher serum INSL3 concentration of peripubertal bulls. Dilution controls showed this to have no effect on the concentration measured. Assay range was 0.02–16 ng/ml; intra- and inter-plate coefficients of variation at the lower, middle and upper range limits were intra: <1% at all concentrations, and inter: 8.0%, 3.3% and 4.4%.

### Statistics

Animals were allocated to the nearest monthly bins based on dates of birth and analysis. Multiple comparisons made use of ANOVA followed by Tukey post hoc tests of significance applying Graphpad Prism, version 7.0 (Graphpad Software, San Diego, CA). For the multiple correlation, Pearson bivariate analysis was used without further correction (SPSS software package, IBM United Kingdom Limited, Portsmouth, UK). Since mean INSL3 values for each treatment group did not differ significantly between time-points after 8 months, where indicated, values were pooled for the period 8–10 months to create a ‘mean INSL3 (8–10 months)’ representative of the maximal concentration achieved in each individual at puberty. Similarly, since these values showed no significant difference in the effect of the peri-pubertal feeding regimen (post 6 months), data were further combined to represent only the differences between the pre-pubertal feeding regimen (indicated as Hi or Lo, respectively). A similar approach was adopted to compare the effect of feeding regimen on time of puberty. All original underlying data are included in two supplemental datafiles (S1 and S2 Datafiles).

## Results

### Effect of nutrition on pubertal parameters

Data for body weight, scrotal circumference and testosterone (S1 Fig) have been previously reported [4] and are re-drawn here to put INSL3 data in context. The data show that the differential achieved in body weight, scrotal circumference and testosterone by offering a high or low plane of nutrition in the pre-pubertal period (<6 months) can be reversed by altering the nutritional regime in the ensuing 6 months. It also emphasizes the high variance observed for the Leydig cell relevant parameter testosterone.

### Effect of nutrition on circulating INSL3

**Fig 1:**
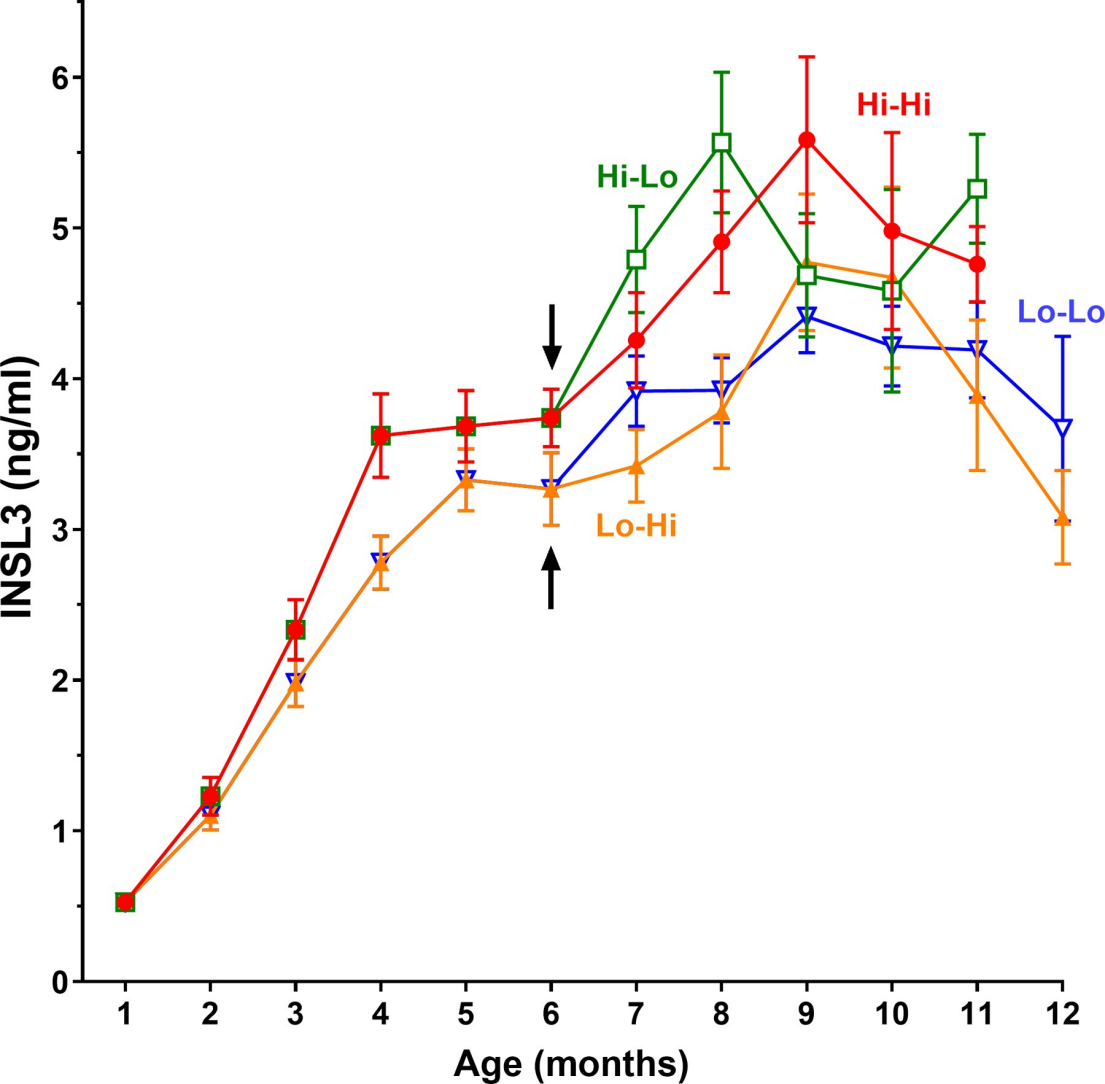
Profiles of INSL3 concentration in peripheral serum during the pre- and peri-pubertal periods. Up to 6 months, Holstein bull calves were fed either a high plane of nutrition (Hi) or a low plane of nutrition (Lo). At 6 months (black arrows) four groups (Hi-Hi, red circles; Hi-Lo, green squares; Lo-Hi, ochre upright triangles; and Lo-Lo, blue inverted triangles) were segregated, reflecting whether they subsequently received a Hi or Lo feeding regimen. Data are given as ng/ml (means ± SEM).

**Fig 2:**
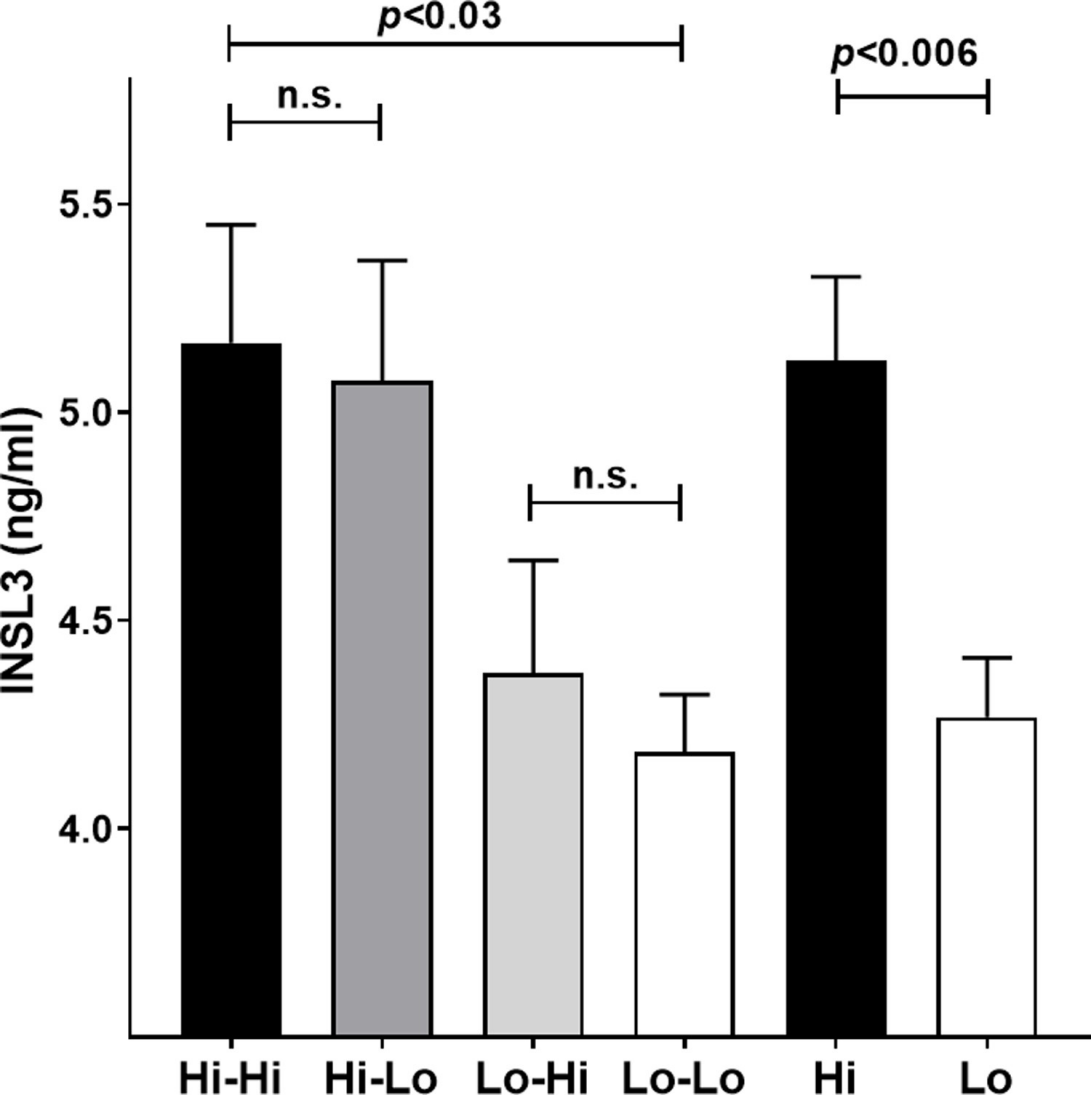
Average INSL3 concentration at months 8 to 10. Holstein bull calves were fed either a high (Hi-Hi) or low (Lo-Lo) plane of nutrition throughout, or had the nutritional paradigm reversed at 6 months to create Hi-Lo or Lo-Hi groups, as indicated. Hi or Lo, represent the combined INSL3 concentration for all animals fed either a high (Hi) or low (Lo) plane of nutrition in the first 6 months, irrespective of the later feeding regimen. Data are given as ng/ml (means ± SEM); n.s., not significant.

INSL3 reflected the development of the Leydig cells in the pre- and peri-pubertal period in dairy bulls (Fig 1), and notably with a relatively smaller variance, compared to the other Leydig cell parameter, testosterone (S1C Fig). Individual INSL3 values ranged from approximately 0.1 ng/ml at 1 month to maximal values of approximately 10 ng/ml at puberty. As for the other parameters (S1 Fig), there was a significant differential effect of the plane of nutrition on circulating INSL3 concentration in the first 6 months (Fig 1), which was statistically significant at most time-points. Importantly, and unlike the other parameters, changing the plane of nutrition in the peri-pubertal period (6–12 months) did not markedly influence this difference (Fig 1). Because the data for the individual groups did not differ significantly between time-points after 8 months, representing the maximal levels achieved at puberty, these have been pooled to provide the results in Fig 2, which illustrate clearly the lack of effect of nutrition on INSL3 in the later peri-pubertal period. Those animals fed a high plane of nutrition in the first 6 months achieved maximal INSL3 concentration at 8 months, whereas those fed a low plane of nutrition in the same period reached maximal INSL3 concentration somewhat later (Fig 1).

### Relationship between INSL3 concentration and pubertal parameters

**Fig 3:**
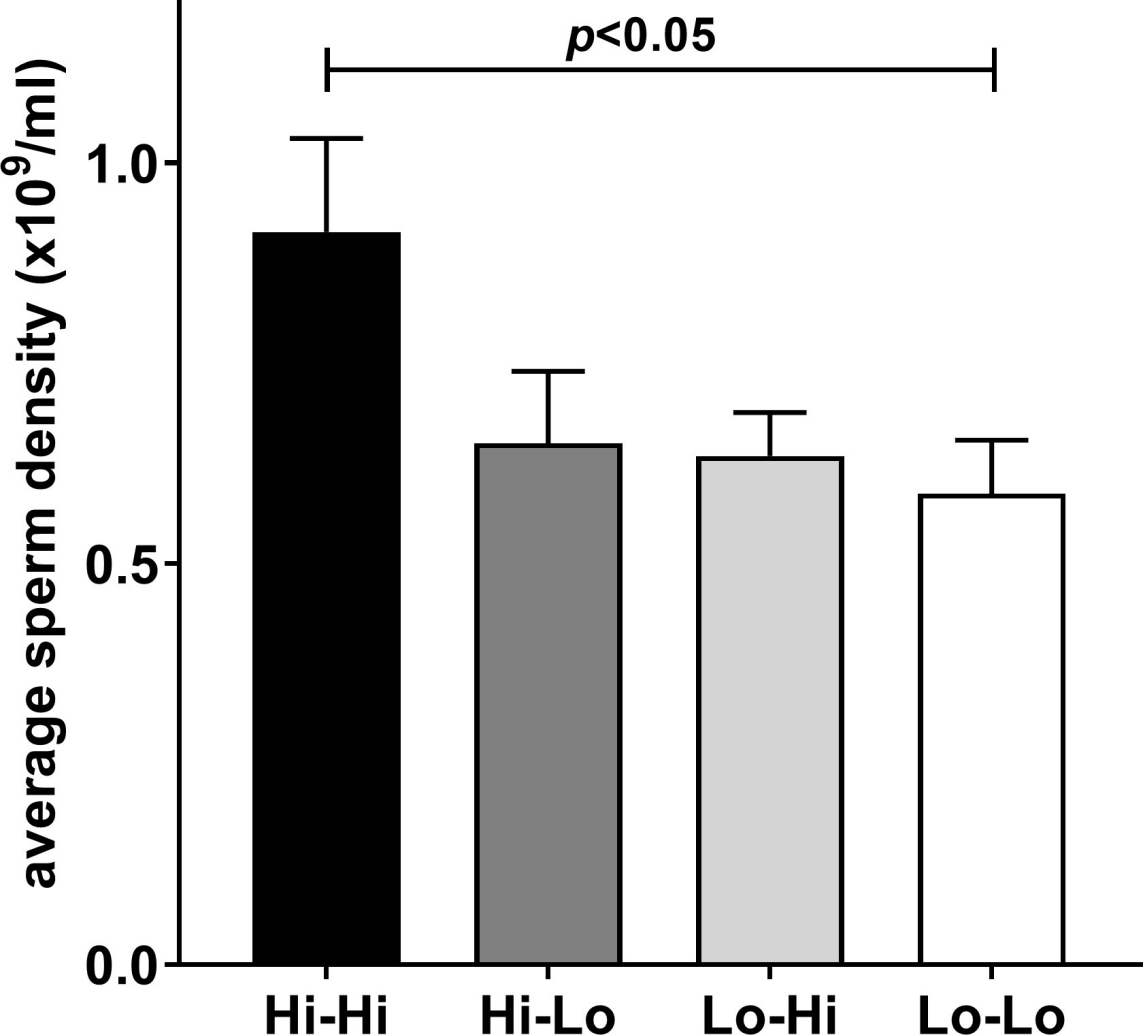
Average sperm density in 4–7 successive ejaculates per animal. Semen samples were collected after puberty between months 12 and 18. Data are given as number of sperm x10^9^/ml (means ± SEM).

**Fig 4:**
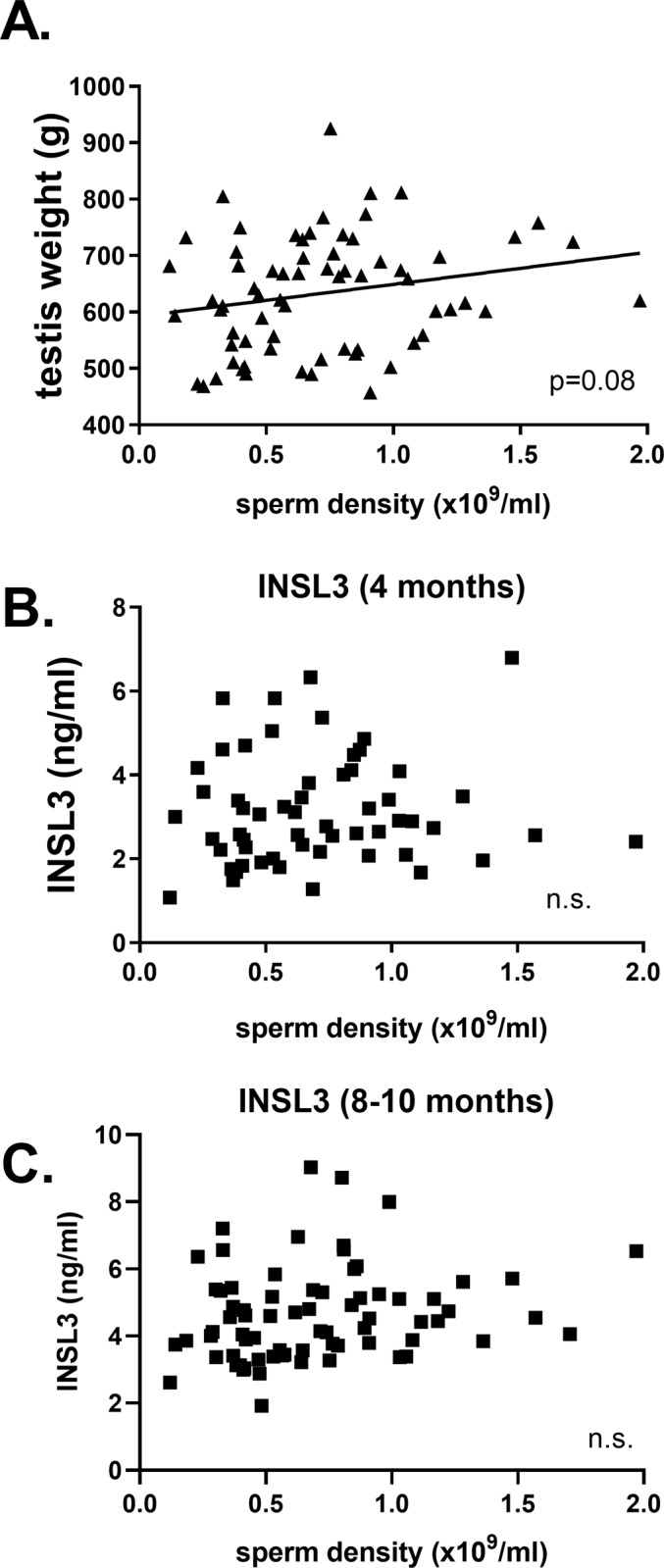
**Regressions of average sperm density against (A) paired testis weight at slaughter (18 months), (B) INSL3 concentration at 4 months, and (C) average INSL3 concentration at 8–10 months)**. Probability of a significant regression is indicated as *p* value, or n.s. (not significant).

**Fig 5:**
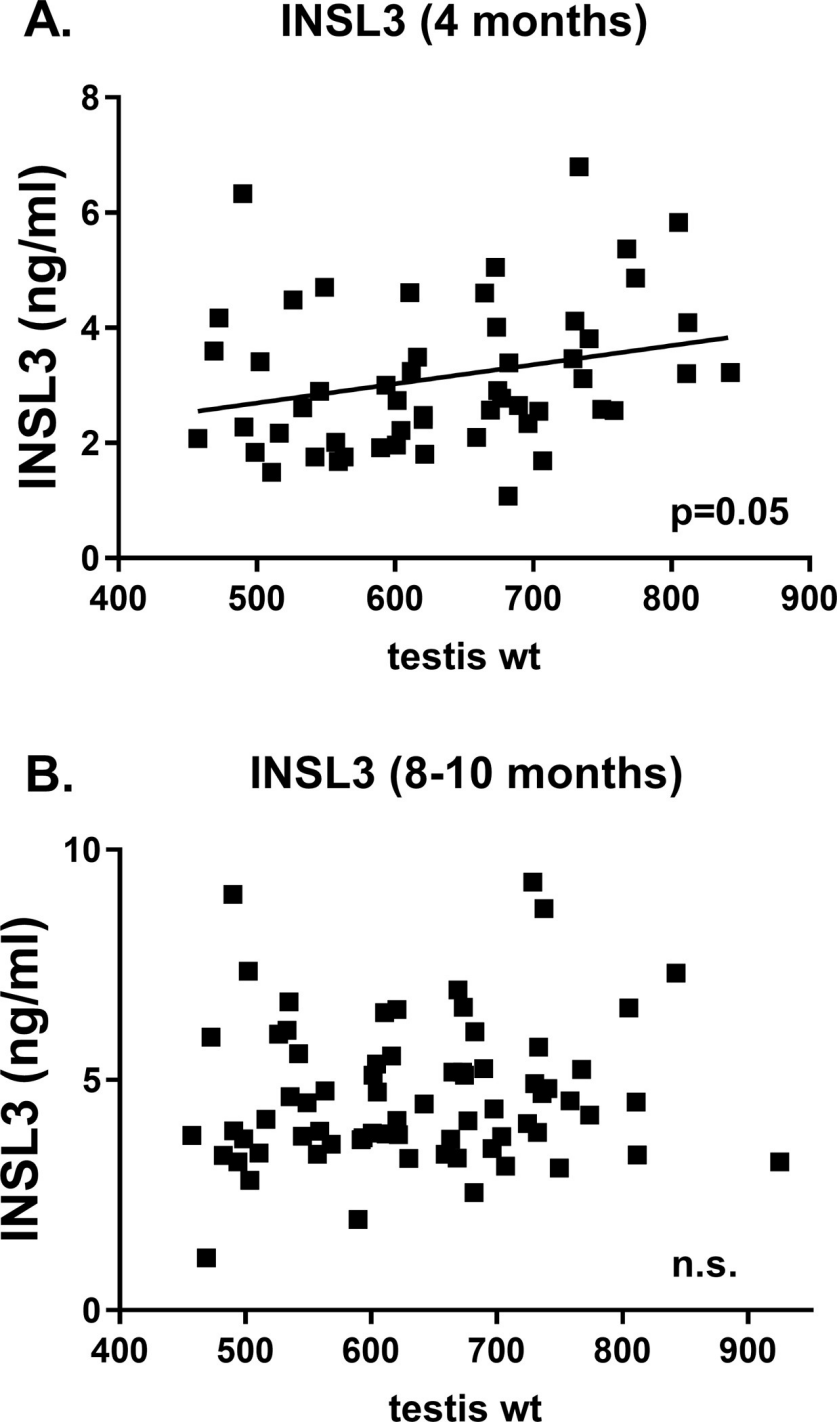
**Regressions of paired testis weight at slaughter (18 months) against (A) INSL3 concentration at 4 months, and (B) average INSL3 concentration at 8–10 months**. Probability of a significant regression is indicated as p value, or n.s. (not significant).

Two parameters which are important from the perspective of commercial semen production are the timing of puberty (defined here as the minimum time in days to have an ejaculate with >50 million sperm and >10% sperm with progressive motility [5]) and the average density of sperm in an ejaculate. Considering the latter parameter, averaging the sperm density for between 4 and 7 successive ejaculates taken once puberty has been achieved (12–18 months) shows that a consistently high plane of nutrition positively influences the sperm density (Fig 3), though any period of low nutrition has a negative effect. Neither testis weight at slaughter (18 months) nor INSL3 concentration appear to correlate with average sperm density (Fig 4A–4C), although the former does exhibit a trend towards significance (p<0.10; Fig 4A). Whereas Fig 4C represents the average INSL3 concentrations in months 8–10, Fig 4B shows values for INSL3 at 4 months, the time for which INSL3 appears to have maximal discriminatory ability in regard to nutritional regimen. This is interesting since INSL3 at 4 months (Fig 5A), though not at 8–10 months (Fig 5B), does appear to correlate with later testis weight at slaughter at 18 months.

**Fig 6:**
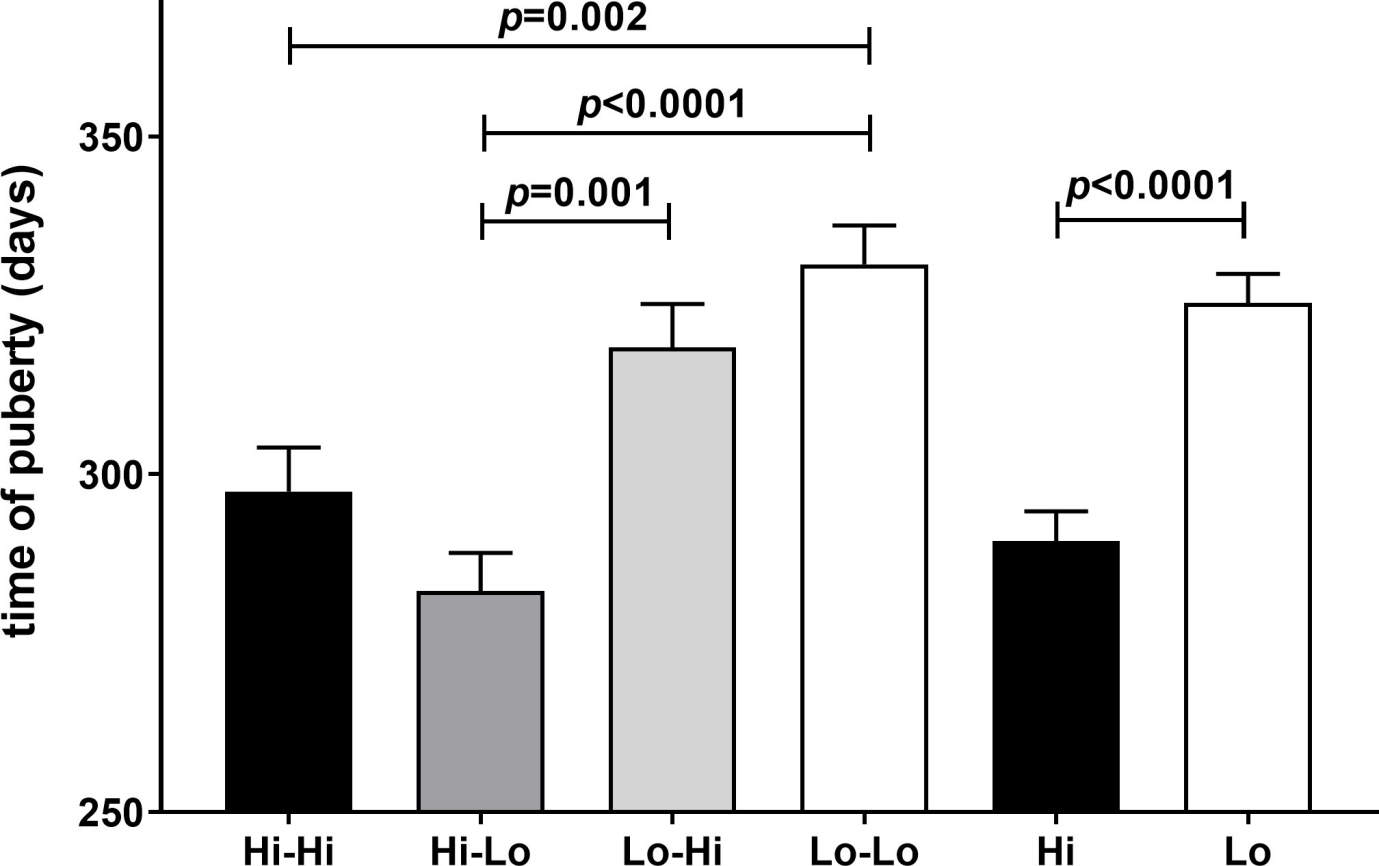
Timing of puberty (days) segregated by nutritional regimen. Puberty is measured as first appearance in the ejaculate of >50 million sperm and >10% forward motility, segregated by nutritional regimen as in Fig 2. Probability of a significant regression is indicated as p value.

**Fig 7:**
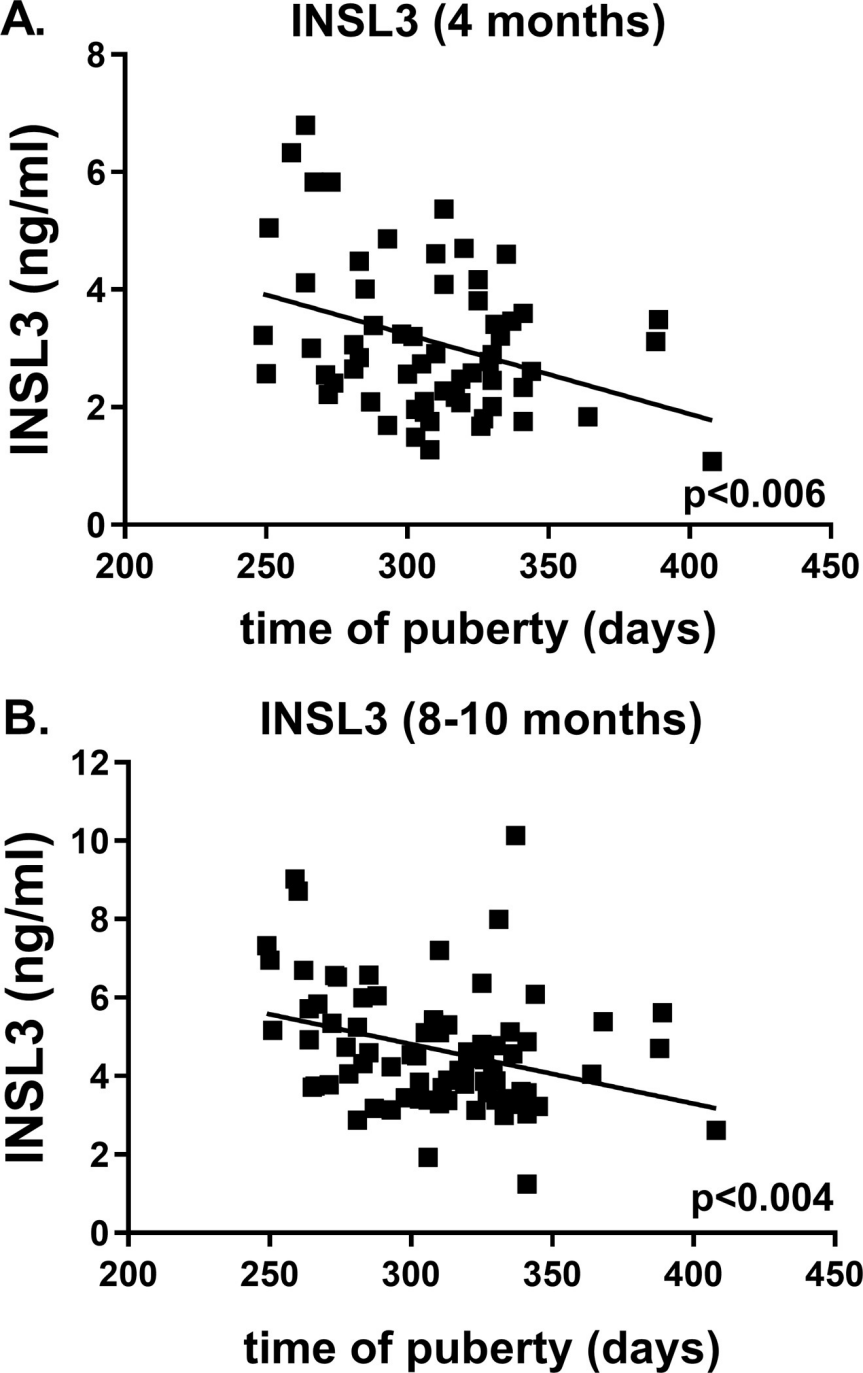
**Regressions of timing of puberty (days) against INSL3 measured at (A) 4 months or (B) 8–10 months**. Probability of a significant regression is indicated as p value.

The timing of puberty in days indicates that nutrition in the prepubertal period is critical, and that this cannot be compensated by improving nutrition in the peri-pubertal period after 6 months (Fig 6) [25]. Importantly, this relationship is very similar to that for INSL3 (Fig 2), and in fact INSL3 either measured in the prepubertal phase at 4 months (Fig 7A) or in the peri-pubertal period at 8–10 months (Fig 7B) indicates a good correlation with the timing of puberty as defined here.

### Multiple correlation analysis

Multiple correlation analysis was undertaken to determine, of those parameters measured, those factors best able to predict either sperm density in the early post-pubertal period or the time to puberty (Table 1). Besides the plane of nutrition and post-pubertal (12 months) scrotal circumference, only INSL3 measured at either 4 months or at puberty, or IGF1, showed a significant correlation. Interestingly, none of the classical hormones of the HPG axis (LH, FSH, testosterone) suggested any predictive ability in regard to sperm production and timing of puberty onset.

**Table 1:** Multiple correlation analysis.

Parameter	Sperm density	Time of puberty	INSL3 (8mo)
	*p-value (Pearson r)*	*p-value (Pearson r)*	*p-value (Pearson r)*
Sperm density (12–18 mo)	-	0.041 (-0.236)	0.036 (0.240)
Time of puberty (days)	0.041 (-0.236)	-	0.000 (-0.395)
Testis weight (18 mo)	Ns	ns	0.046 (0.238)
Plane of nutrition (<6 mo)	0.032 (-0.246)	0.000 (0.535)	0.000 (-0.408)
Plane of nutrition (>6 mo)	0.008 (-0.303)	0.000 (0.482)	0.004 (-0.317)
Scrotal circumference (12 mo)	0.005 (0.326)	0.000 (-0.553)	0.005 (0.314)
INSL3 (8–10 mo)	Ns	0.001 (-0.359)	0.000 (0.836)
INSL3 (4 mo)	ns	0.006 (-0.351)	0.001 (0.415)
INSL3 (8 mo)	0.036 (0.240)	0.000 (-0.395)	-
LH (4 mo)	ns	ns	ns
LH (8 mo)	ns	ns	ns
FSH (4 mo)	ns	ns	ns
FSH (8 mo)	ns	ns	ns
Testosterone (4 mo)	ns	ns	ns
Testosterone (8 mo)	ns	ns	ns
IGF1 (4 mo)	ns	0.001 (-0.367)	0.000 (0.395)
IGF1 (8 mo)	0.003 (0.341)	ns	ns

Data for individual animal parameters (left column) were subjected to multiple correlation analysis against average sperm density, time of puberty, and INSL3 concentration at 8 months. Only significance values *p*<0.05 are indicated. ns, not significant. 0.000, p<0.0005.

## Discussion

INSL3 has been shown to be an accurate measure of Leydig cell development during male puberty in several mammals, including rodents [16], humans [32–34], pigs [35] and ruminants, such as goats [36] and bulls [24,37]. Its value lies both in its more or less constitutive expression pattern, with low intra-individual biological variance, the accuracy of modern assays, with low technical variance, and its significance in effectively measuring Leydig cell functional capacity [2]. It therefore differs substantially from other Leydig-related parameters such as testosterone or LH, or their derivatives, such as the testosterone/LH ratio, with their large biological and/or technical variance. Leydig cell functional capacity can be considered as the product of cell number and their differentiation status, and thus represents their potential to produce testicular steroids as well as non-steroidal pro-spermatogenic factors. In most mammals, other than seasonal breeders, the Leydig cells undergo a phase of proliferation from resident stem cells during pre-puberty, followed by differentiation, which results in a fixed number of Leydig cells persisting with only modest attrition throughout life [1]. Only where there is a specific involution, or Leydig cell death, as may occur in seasonal breeders [2,38], or subsequent to a steroidal contraceptive regimen, or when rats are treated with the toxicant ethane dimethyl sulfonate, is there evidence for a regeneration of Leydig cells from resident peritubular stem cells [39]. Leydig cell mitosis in the normal adult testis of any mammal is an extremely rare event. Importantly, by measuring Leydig cell functional capacity, INSL3 has been shown, for example in rats, to reflect accurately the development of the HPG axis through puberty [19]. Commencing at low levels in the postnatal period, circulating INSL3 increases during pubertal development as the pulses of LH increase in frequency and amplitude, concomitant with Leydig cell proliferation and subsequent differentiation; it reaches a zenith at puberty when Leydig cells have stopped proliferating, sperm are first evident in the epididymis, and both LH and testosterone pulses are maximal. INSL3 concentration then declines somewhat to a stable adult concentration with the attainment of regular sperm and steroid production in young adulthood [16].

Functionally, INSL3 reflects Leydig cell differentiation and hence steroidogenesis and the production of androgens essential for Sertoli cell function and spermatogenesis [1]. INSL3 also directly impacts on spermatogenesis, via its specific receptors, called RXFP2 (relaxin family peptide receptor 2), which are expressed on pre- and post-meiotic male germ cells [40]. Studies in rats and boars have shown that a lack of INSL3 leads to increased germ cell apoptosis [41,42], and in a male steroidal contraception study in humans residual spermatogenesis was greatest where there was highest circulating INSL3 [43]. Therefore, although Leydig cells comprise less than 10% of the testis, INSL3 production (though not testosterone) in bulls correlates with sperm production, scrotal circumference, and testis weight [24] (Table 1). Moreover, INSL3 concentration is reduced in bulls where there appears to be deficient spermatogenesis [23,37]. Because of its low biological and technical variance, INSL3 appears to be better for monitoring testis development than either LH or testosterone, both of which are highly pulsatile. Although scrotal circumference also correlates well with the timing of puberty and sperm density in the present study, there are several factors which in practise can confound its application; these include the thickness of subdermal scrotal fat, as well as common pathological conditions including scrotal edema [44,45].

This is the first study to use INSL3 in the assessment of puberty in dairy bulls. Previous studies have looked at puberty in Japanese Black beef bulls [21,23,37], as well as in goats [36]. The present study also exploited the high resolution of INSL3 as a puberty parameter to look at the effect of pre- or peri-pubertal feeding regimen on pubertal development. Scrotal circumference (S1B Fig), body weight (S1A Fig) and IGF1 [4,25] were greater in the prepubertal period with a high plane of nutrition promoting the parameters, compared to a low plane of nutrition. This effect was mostly reversed when the treatments were exchanged in the peri-pubertal period between 6 and 12 months (S1 Fig) [4]. A similar finding was noted also for testosterone (S1C Fig) though, because of the large parameter variance, differences between treatments were not significant in the peri-pubertal period. Importantly, the timing of puberty as defined by the presence of >50 million sperm in the ejaculate was not reversed by exchanging treatments after 6 months [4] (Fig 6) but followed largely the feeding regimen from the first 6 months. Total sperm counts appeared not to show any marked influence of diet [4], once puberty had been attained. To reduce the variance in this parameter, we also averaged the sperm concentration in ejaculates for between 4 and 7 successive electroejaculations between 12 and 18 months within any one animal, thus more reflecting the attainment goal of commercially suitable ejaculates as early as possible after puberty. With this parameter, we observed that the Hi-Hi feeding regimen was superior to the other three feeding combinations (Fig 3). Applying this parameter in other data comparisons showed that both scrotal circumference (12 months) and the average INSL3 concentration at 8 months were predictive of post-pubertal average sperm density (Table 1).

The key parameter to show the benefits of dietary choice on improving sperm harvesting potential, appears to be the timing of puberty itself, which is advanced by approximately 1 month consequent on feeding a high plane of nutrition in the prepubertal phase up to 6 months (Fig 6). Interestingly, of the parameters measured, INSL3 shows one of the closest correlations with the timing of puberty and is also predictive of total testis weight at 18 months. INSL3 was also shown to correlate closely with total sperm count and testis weight in Japanese Black beef bulls kept on a single feeding regimen [24]. Importantly, if we select the peripheral INSL3 concentration at 4 months, which represents the time within the prepubertal period when the INSL3 profile is increasing most rapidly and consequently shows most discriminatory ability, then this value (and unlike other parameters, except for IGF1) is highly predictive not only of the timing of puberty (Fig 7A), but also of later scrotal circumference, though not of later testis weight nor average sperm density (12–18 months). In an independent study, INSL3 mRNA in the testes of calves slaughtered at a comparable time-point (18 weeks) was also shown to be significantly reduced if the calves were fed a low plane of nutrition [46]. IGF1 is known to reflect growth potential (and hence is a logical readout of improved nutritional status) and is also an important co-factor in the expression of numerous gonadally expressed genes [47].

## Concluding remarks

In conclusion, this study reinforces the importance of offering a high plane of nutrition in the first 6 months in order to advance sperm production in dairy bulls, and that the Leydig cell parameter INSL3 closely predicts this effect. This implies that INSL3 is not only representing Leydig cell development during puberty, but also, through the direct and/or indirect effects of Leydig cell function on spermatogenesis, appears to reflect sperm production as well.

## Supporting information

S1 Fig **Profiles of (A) body weight, (B) scrotal circumference, and (C) serum total testosterone during the pre- and peri-pubertal periods.** Up to 6 months, Holstein bull calves were fed either a high plane of nutrition (Hi) or a low plane of nutrition (Lo). At 6 months (black arrows) four groups (Hi-Hi, red circles; Hi-Lo, green squares; Lo-Hi, ochre upright triangles and Lo-Lo, blue inverted triangles) were segregated, reflecting whether they subsequently received a Hi or Lo feeding regimen. Data are given as means + SEM; based on data presented in [4,25].
(PDF)

S1 Datafile (XLSX)

S2 Datafile (XLSX)
