# Supplementary figures and images for: Prepubertal nutrition alters Leydig cell functional capacity and timing of puberty
Source: PLoS One. 2019 Nov 21;14(11):e0225465. doi: 10.1371/journal.pone.0225465 (PMC6872131; doi:10.1371/journal.pone.0225465)

S1\_Fig

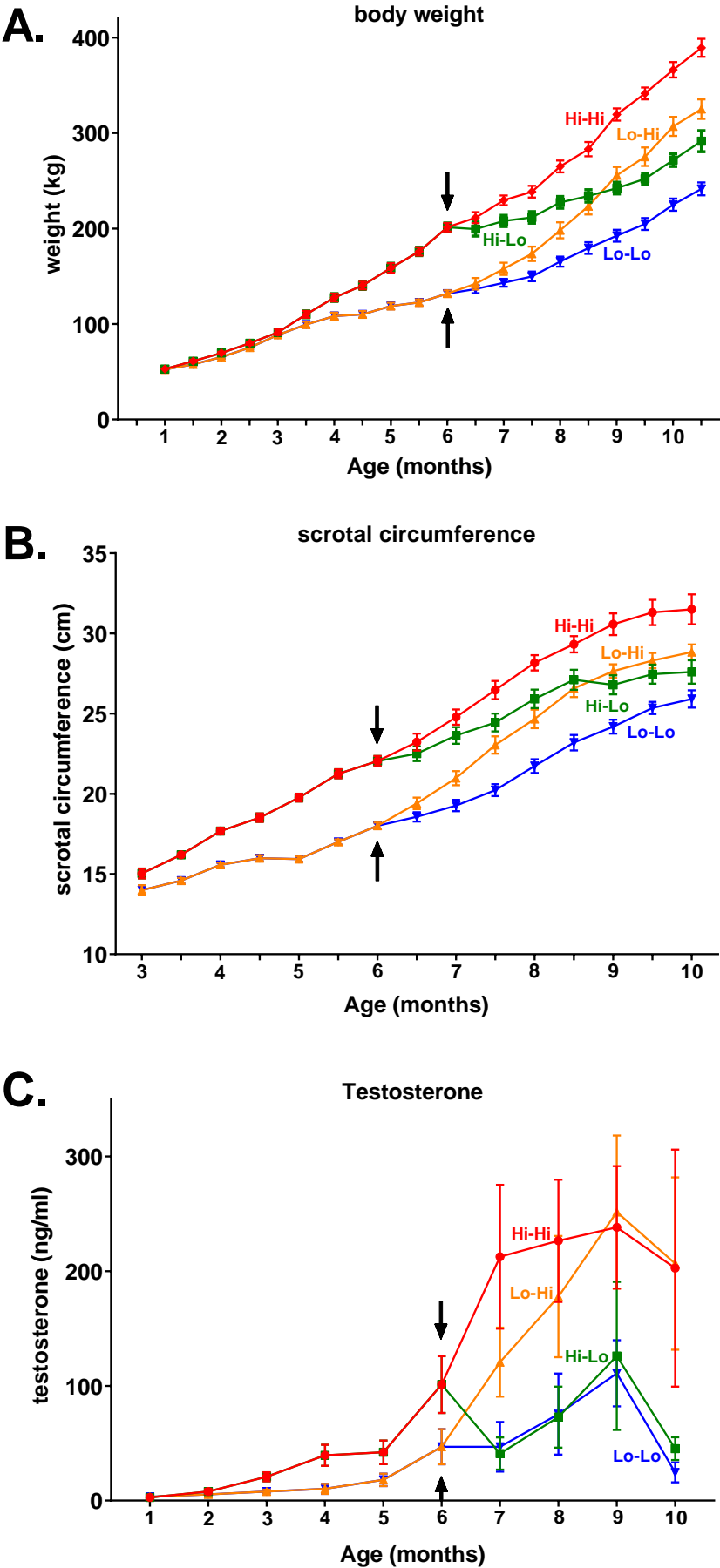

Supplement: S1 Fig — Profiles of (A) body weight, (B) scrotal circumference, and (C) serum total testosterone during the pre- and peri-pubertal periods. Up to 6 months, Holstein bull calves were fed either a high plane of nutrition (Hi) or a low plane of nutrition (Lo). At 6 months (black arrows) four groups (Hi-Hi, red circles; Hi-Lo, green squares; Lo-Hi, ochre upright triangles and Lo-Lo, blue inverted triangles) were segregated, reflecting whether they subsequently received a Hi or Lo feeding regimen. Data are given as means + SEM; based on data presented in [4,25]. (PDF) [file pone.0225465.s001.pdf]
